# Using fundamental knowledge of induced resistance to develop control strategies for bacterial canker of kiwifruit caused by *Pseudomonas syringae* pv. *actinidiae*

**DOI:** 10.3389/fpls.2013.00024

**Published:** 2013-02-22

**Authors:** Tony Reglinski, Joel L. Vanneste, Kirstin Wurms, Elaine Gould, Francesco Spinelli, Erik Rikkerink

**Affiliations:** ^1^The New Zealand Institute for Plant and Food Research LimitedHamilton, New Zealand; ^2^Department of Agricultural Sciences, University of BolognaBologna, Italy

*Pseudomonas syringae* pv. *actinidiae* (Psa) which causes bacterial canker of kiwifruit (*Actinidia deliciosa* and *A. chinensis*) was first isolated in Japan in 1984 (Takikawa et al., 1989), and soon after in Korea (Koh et al., 1994) and Italy (Scortichini, 1994). The economic impact on the global production of kiwifruit of those early occurrences was relatively limited (Vanneste et al., 2011). However, the latest outbreak of Psa which started in Italy in 2008 and rapidly spread throughout most of the kiwifruit growing regions of the world, represents a major threat to the global kiwifruit industry (Vanneste, 2012). The pathovar *actinidiae* is not a genetically homogeneous pathovar; strains can be grouped in four biovars based on their molecular, microbiological and pathogenic characteristics (Vanneste et al., 2013) which is consistent with MLST and whole genome sequence analysis (Ferrante and Scortichini, 2010; Mazzaglia et al., 2011; Chapman et al., 2012). The recent outbreak of bacterial canker on kiwifruit in Europe and New Zealand is caused by the same biovar of Psa (biovar 3) (Chapman et al., 2012; Vanneste et al., 2013). During the 2 years that the pathogen has been present in New Zealand, over 60% of the area planted in kiwifruit has been affected (Kiwifruit Vine Health, 2012). This rapid spread may be attributable to the virulence of biovar 3 and to the scarcity of products available for control of plant pathogenic bacteria in general, and Psa in particular. Many products used for control of plant pathogenic bacteria contain antibiotics (mostly streptomycin) or heavy metals (mostly copper). Both types of products do have limitations because of phytotoxicity or because they are not authorized in some countries (e.g., antibiotics in Europe). This has led to a large screening programme in New Zealand for the identification of potentially effective products to control Psa. The products tested included a number of commercially available potential elicitors of host resistance. One of the most effective elicitors in glasshouse trials on *A. chinensis* and *A. deliciosa* was acibenzolar-S-methyl [ASM], sold under the names of Bion® or Actigard® (Syngenta).

ASM belongs to the benzothiadiazole chemical group and operates as a functional analogue of salicylic acid. It has demonstrated good efficacy against bacterial diseases, including bacterial spot *(Xanthomonas axonopodis* pv. *vesicatoria*) and bacterial speck *(P. syringae* pv. *tomato*) in tomato (Louws et al., 2001), fire blight (*Erwinia amylovora*) in apples (Bastas and Maden, 2007), pear (Spinelli et al., 2006) and quince (Bastas and Maden, 2007), and xanthomonas leaf blight (*X. axonopodis* pv. *allii*) in onions (Gent and Schwartz, 2005). However, while elicitors can be very effective in controlled conditions, the host response can be highly variable in the field, thus raising questions about their potential for disease management. Furthermore, there is evidence that induced resistance, whether via the use of chemical elicitors or by constitutive expression of inducible defenses, can be accompanied by reduced fruit production and/or quality (Walters and Heil, 2007; Cipollini and Heil, 2010). These observations are consistent with the theory that induced resistance evolved as a strategy to minimize the metabolic costs associated with defense (Karban, 2011). Plant genotype and environment factors can also affect the relative benefits and costs of induced resistance (Cipollini and Heil, 2010; Walters et al., 2011) and a greater understanding of these dynamic interactions is necessary to facilitate more effective use of elicitors for disease control.

**Figure 1:**
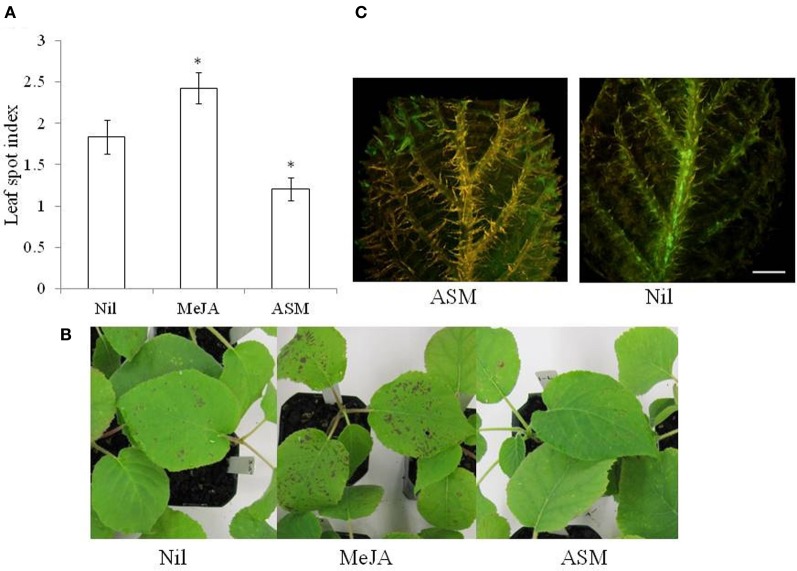
**(A,B)** Effect of foliar spray with 1.7 mM acibenzolar-S-methyl (ASM) and 1.1 mM methyl jasmonate (MeJA) on Psa infection in *Actinidia chinensis* **(A,B)**. Treatments were applied 1 week before spray inoculation with a suspension containing 10^9^ cfu ml^−1^ of *Pseudomonas syringae* pv. *actinidiae* (strain 10627). Plants were assessed 2 weeks later and the leaf spotting was recorded according to the following index, 0 = 0% leaf area, 1 ≤ 10%, 2 = 10–25%, 3 = 25–50%, 4 ≥ 50%. The data are presented as means ± standard error (*n* = 9) and the asterisk indicates a significant difference between the treatment and the untreated control (LSD = 0.53, *P* < 0.05). **(C)** Fluorescent stereomicroscopy of *A. deliciosa* leaves inoculated with GFPuv labeled Psa (strain CFBP7286). Inoculation was performed by cutting the leaf tip with scissor dipped in a bacterial suspension (10^9^ cfu ml^−1^). The photos were taken 2 weeks after inoculation. Measuring bar = 2 mm.

Complementary studies that target both fundamental and applied aspects of plant innate immunity are critical to realize the potential of induced resistance. Typically, inducible defenses are triggered upon recognition of pathogen-derived molecules. These molecules were historically termed elicitors or avirulence factors, but have more recently been renamed microbe-associated molecular patterns (MAMPs) and effectors, respectively (Jones and Dangl, 2006; Bent and Mackey, 2007). Phytohormone-mediated signaling pathways play a key role in orchestrating the plant response, with cross-talk between salicylic acid (SA), jasmonic acid (JA), and ethylene (ET) pathways providing means whereby the plant can tailor its defense response to different pathogens and pests (Robert-Seilaniantz et al., 2011; Pieterse et al., 2012). The SA and JA/ET defense pathways are often mutually antagonistic. However, synergistic interactions have been reported in some pathosystems (Pieterse et al., 2009). Abscisic acid (ABA) has also been shown to interact with defense-signaling pathways and it is proposed that ABA operates as a global regulator and co-ordinates the plant response to simultaneous multiple stresses (Ton et al., 2009). ABA-regulated stomatal closure is a key element of pre-invasion SA-regulated innate immunity to *P. syringae* in *Arabidopsis* (Melotto et al., 2006) and therefore its role in the kiwifruit/Psa interaction is of interest given that glasshouse studies indicate that kiwifruit resistance to Psa is mediated via the SA signaling pathway. Incidence of the disease was significantly decreased (*p* < 0.05)_on *A. chinensis* seedlings previously treated with ASM as a foliar application (spray) while a significant increase in disease was observed on plants treated with methyl jasmonate (Figure 1). Moreover, histological evidence suggests that the pathogen is less able to colonize ASM-treated leaves than untreated leaves (Spinelli et al., 2011).

The number of tools available to analyse and probe the relationships between these host response pathways has grown considerably in recent times. In addition, the affordability of techniques such as Next Generation Sequencing (NGS) has improved considerably and, as a result, these tools can now be applied to many different situations. Increasingly these tools are helping us to understand the suite of genes affected by biotic and abiotic elicitors and the host response associated with major gene resistance (e.g., Kim et al., 2011; Gyetvai et al., 2012). Inevitably, some of the genes involved in these responses are in common, allowing researchers to look for potential synergy or antagonism between these responses. To increase our understanding, we are employing several molecular tools including: (1) NGS to measure total RNA expression in response to time and application of different elicitors on different cultivars; (2) quantitative PCR (qPCR) to study in depth the responses of putative resistance and defense response genes that have already been shown to play a role in kiwifruit interactions with other pests and diseases (Wurms et al., 2011a,b), (3) gene mining of the extensive database of the kiwifruit genome (Crowhurst et al., 2008) to identify novel gene candidates for study, and (4) transformation studies involving up- or down-regulation of specific genes of interest to assess their roles in the kiwifruit-Psa interaction. To date, our qPCR studies on a small set of candidate genes have identified several transcripts that are induced by Psa on its own and by ASM on its own. Moreover, the expression of these genes is enhanced further when ASM-treated plants are inoculated with Psa; this response correlates with decreased disease expression and is consistent with the phenomenon of priming whereby elicitor-treated plants react more rapidly and/or strongly to pathogen attack (Conrath, 2009). Up-regulated genes in this qPCR study included phenylalanine ammonia lyase (PAL), a key regulatory enzyme in the production of antimicrobial phytoalexins (Naoumkina et al., 2010), a hypersensitivity-induced response protein, a protein that interacts with putative plant R genes (Jung and Hwang, 2007; Jung et al., 2008), and RIN4—a protein thought to play a key role in defense against bacterial pathogens such as *Pseudomonas* spp., and which is involved in both MAMP-triggered and effector-triggered immunity (Afzal et al., 2011).

The analysis of plant immunity in Arabidopsis and tomato model systems has provided basic knowledge of pathogen virulence factors (e.g., effectors), the host proteins/pathways targeted by some of these virulence factors, and how manipulation events are detected by major resistance genes (R genes) (Jones and Dangl, 2006; Dangl, 2007; Nishimura and Dangl, 2010; Schwessinger and Ronald, 2012). This information has been instrumental in shaping the current study by identifying potential targets that can be examined in the context of the Psa-kiwifruit interaction. It is suggested that pathogen effectors might converge on a limited set of host proteins with important regulatory roles in plant immune signaling (Mukhtar et al., 2011; Spoel and Dong, 2012). Psa contains several effectors (Marcelletti et al., 2011) that are known to interfere with RIN4 in the model pathosystem *Pseudomonas*-*Arabidopsis*. AvrRPM1 is known to induce phosphorylation of RIN4 (Mackey et al., 2002), while HopF2 interferes with the resistance triggered by RIN4s interaction with another effector-AvrRpt2 (Wilton et al., 2010). The exact mechanism of these interactions is not yet understood. Our genome analysis has also identified other candidates that possibly interfere with this protein, such as a distantly related member of the AvrRPM1 effector family (AvrRPM2). It is likely that RIN4 is not the only host target of Psa and the use of NGS and other approaches may identify additional host targets affected by ASM and/or Psa. Further expression studies by qPCR, NGS, and transformation studies will determine whether expression of these genes can be used as a marker in breeding and/or elicitor selection. Other tools, such as yeast 2-hybrid and *in planta* protein-protein interaction screening tools, are also being employed to decipher how pathogen and host proteins interact. Together this new knowledge may also allow us to fine tune elicitor-based strategies (e.g., delivery, timing, and frequency) in order to maximize their impact for the control of plant disease.

The project combines applied and fundamental research to identify methods to protect commercial kiwifruit production from the threat posed by Psa. By integrating these approaches, we can harness the true potential of elicitors both to protect existing kiwifruit cultivars and to develop new cultivars with increased resistance to Psa. For example, as our knowledge about the targets of effectors increases, so will our understanding of which of these targets are involved in other pertinent host pathways, e.g., in response to elicitors or plant hormones. As these effectors are also key components of recognition by R genes, this should allow us to postulate both favorable and unfavorable interactions between elicitor-induced pathways and certain R gene strategies. As the effectors AvrRPM1 and HopF2 both target RIN4, and RIN4 RNA expression appears to be affected by ASM elicitation, there is potential for the perturbation of resistance responses that rely on these effectors by ASM. Depending on the magnitude of the ASM effect on the amount of RIN4 protein, and the nature of the molecular mechanisms involved, the end result could either be neutral, beneficial to, or detrimental to such a resistance response. This simple example illustrates how future fundamental research is needed to reveal the nature of these mechanisms, and to complement resistance breeding and crop protection strategies.
